# Programmable Ligand Detection System in Plants through a Synthetic Signal Transduction Pathway

**DOI:** 10.1371/journal.pone.0016292

**Published:** 2011-01-25

**Authors:** Mauricio S. Antunes, Kevin J. Morey, J. Jeff Smith, Kirk D. Albrecht, Tessa A. Bowen, Jeffrey K. Zdunek, Jared F. Troupe, Matthew J. Cuneo, Colleen T. Webb, Homme W. Hellinga, June I. Medford

**Affiliations:** 1 Department of Biology, Colorado State University, Fort Collins, Colorado, United States of America; 2 Department of Biochemistry, Duke University Medical Center, Durham, North Carolina, United States of America; Leeds Institute of Molecular Medicine, United Kingdom

## Abstract

**Background:**

There is an unmet need to monitor human and natural environments for substances that are intentionally or unintentionally introduced. A long-sought goal is to adapt plants to sense and respond to specific substances for use as environmental monitors. Computationally re-designed periplasmic binding proteins (PBPs) provide a means to design highly sensitive and specific ligand sensing capabilities in receptors. Input from these proteins can be linked to gene expression through histidine kinase (HK) mediated signaling. Components of HK signaling systems are evolutionarily conserved between bacteria and plants. We previously reported that in response to cytokinin-mediated HK activation in plants, the bacterial response regulator PhoB translocates to the nucleus and activates transcription. Also, we previously described a plant visual response system, the de-greening circuit, a threshold sensitive reporter system that produces a visual response which is remotely detectable and quantifiable.

**Methodology/Principal Findings:**

We describe assembly and function of a complete synthetic signal transduction pathway in plants that links input from computationally re-designed PBPs to a visual response. To sense extracellular ligands, we targeted the computational re-designed PBPs to the apoplast. PBPs bind the ligand and develop affinity for the extracellular domain of a chemotactic protein, Trg. We experimentally developed Trg fusions proteins, which bind the ligand-PBP complex, and activate intracellular PhoR, the HK cognate of PhoB. We then adapted Trg-PhoR fusions for function in plants showing that in the presence of an external ligand PhoB translocates to the nucleus and activates transcription. We linked this input to the de-greening circuit creating a detector plant.

**Conclusions/Significance:**

Our system is modular and PBPs can theoretically be designed to bind most small molecules. Hence our system, with improvements, may allow plants to serve as a simple and inexpensive means to monitor human surroundings for substances such as pollutants, explosives, or chemical agents.

## Introduction

It is currently impractical to monitor most human environments and large areas for the presence of pollutants, explosives, or chemical agents [1]. Living organisms constantly monitor their environment with detection abilities that exceed our current technologies. One strategy to use and expand upon the abilities of living systems to detect substances is to computationally re-design biological receptors that mediate responses to external stimuli. Periplasmic binding proteins (PBPs), normally involved in bacterial chemotaxis, have been computationally re-designed to produce highly specific and sensitive bio-sensing capacities for substances such as explosives, a chemical agent surrogate, and a metal [2], [3], [4], [5]. They have a theoretical capacity to detect any compound that can fit within the protein's binding pocket. Unfortunately, these computationally re-designed proteins are unstable *in vitro*, preventing use of this powerful technology in electronic detectors [3], [6]. These receptors have been used in bacterial biosensors, where re-designed PBPs were linked to gene expression through a histidine kinase signaling pathway [5]. Because it is unrealistic to place living bacteria throughout human environments or over wide areas, computationally re-designed receptors have not found application in detection systems.

Plants have natural detection abilities and are found in most human environments. These properties, along with their ability to cover wide areas, means plants could serve as inexpensive detectors provided they can be made to sense specific substances with high sensitivity and respond in an easily observable manner. We have previously described a plant response system that produces white plants when a “de-greening gene circuit” is transcriptionally induced with an estrogen-like hormone [7]. This de-greening gene circuit provides a visual response or readout that is readily recognized, remotely detectable, relatively rapid (less than two hours), and resettable [7]. If the computer re-designed receptors could be adapted in plants so that their highly specific and sensitive detection abilities are functionally linked to such a plant response, a powerful and inexpensive detection technology would result.

Developing a plant-based detection system based on bacterial PBPs requires detailed understanding of both the natural function of chemotactic PBPs as well as prokaryotic and eukaryotic signal transduction. When an extracellular ligand binds a PBP (e.g., ribose binding protein, RBP) a conformational change is produced leading to increased affinity of the PBP-ligand complex for the extracellular domain of a bacterial chemotactic receptor (e.g., Trg). The periplasmic portion of Trg has previously been fused to the cytoplasmic portion of a histidine kinase (HK), EnvZ, producing the functional chimeric protein Trz [8]. This chimeric Trg-HK protein allows input from the re-designed receptors to be linked to gene expression [5]. Interaction of the receptor-ligand complex with Trg activates the cytoplasmically-localized histidine kinase (EnvZ) through the mutually conserved HAMP domain [8]. The activated HK then initiates an intracellular phospho-relay that regulates gene expression. To enable such a system in plants, the re-designed receptors need to be targeted extracellularly and we need to address eukaryotic processes that are not fully understood, such as transmembrane signaling, signal-dependent nuclear translocation, and other signaling complexities.

We show that evolutionarily conserved histidine kinase signal transduction components [9], [10], [11] can be used to build a synthetic signal transduction system, and we then use this system to link input from computationally re-designed receptors to a plant response. Transmembrane fusions were tested for function in bacteria followed by adaptation for plants. We then show that an adapted HK functions in plants by linking it to a transcriptional response through signal-dependent nuclear translocation of a bacterial response regulator [12], to produce a synthetic signal transduction system. Our synthetic signal transduction pathway is able to functionally link input from the computationally re-designed receptors to a plant response system, producing a prototype detector plant. Further refinements of this system may allow plants to be used as simple and inexpensive detectors of pollutants, explosives and terrorist agents.

## Results

### Targeting the computationally re-designed receptor to the apoplast

PBPs are normally located in the bacterial periplasmic space. To function in plants, re-designed bacterial PBPs must be localized outside the plant cell to sense extracellular ligands, but be retained in such a way that they are able to activate transmembrane HKs. Natural and re-designed PBPs are small proteins; e.g., the receptor for TNT (2,4,6-trinitrotoluene) is 4×7×8 nm in size [5] and thus should freely diffuse in the plant apoplast, which allows unrestricted movement of small proteins (less than approximately 50 nm) [13]. To test whether bacterial PBPs can be targeted to the apoplast, we fused a plant secretory sequence (ss) to the N-terminus of ribose binding protein (RBP), a starting PBP for re-designed receptors, and green fluorescent protein (GFP) and show the fusion protein, ss-RBP-GFP, is localized in the apoplast (Figure S1).

### Design and test of functional signal transduction components in bacteria for a plant synthetic pathway

**Figure 1:**
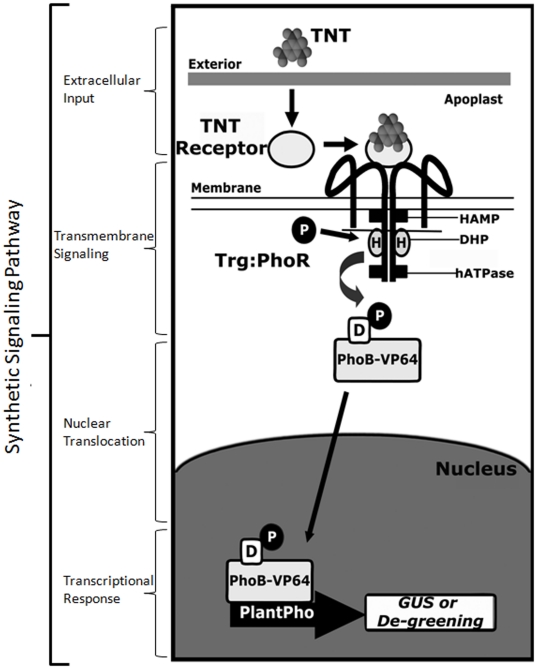
Diagram of synthetic signal transduction system encoded by gene circuits. Diagram of the complete synthetic signal transduction system in transgenic plants, using TNT as the ligand, signaling through the bacterial chimeric histidine kinase, and adapted response regulator, then activating a transcriptional response. ssTNT.R3 → Fls-Trg-PhoR → PhoB-VP64 → *PlantPho* promoter:: GUS or de-greening circuit. *D*, *H*, aspartate and histidine residues; *P*, phosphate; *HAMP*, *DHP* and *hATPase* refer to functional domains of Trg-PhoR [11]. The horizontal line on the intracellular portion of the HK molecule indicates the approximate location of the Trg-PhoR fusion.

In bacteria, the ligand-PBP complex develops high affinity for transmembrane proteins such as Trg, and the chimeric Trg-HK protein allows activation of gene expression with one additional protein, a bacterial response regulator [5]. We previously showed that the bacterial response regulator PhoB [14], [15] can be adapted to function in a partial synthetic eukaryotic signal transduction system [12]. The system is partially synthetic as it requires input from plant HK components (e.g., HKs, histidine phospho-transferases) that are typically activated by cytokinins. In bacteria, PhoB normally accepts a signal from its cognate HK, PhoR [14]. Hence, we could link input from the computationally re-designed PBPs to our partial signal transduction system if a transmembrane signaling protein can be produced with Trg (to bind the ligand-PBP complex) and PhoR to activate PhoB-VP64 (Figure 1). PhoR normally functions in phosphate sensing and lacks the conserved HAMP domain [14], therefore use of a previously established Trg-HK fusion point [8] was not possible.

Because mechanisms involved in transmembrane HK activation are not fully understood, we constructed an experimental system to rationally test multiple fusion points in bacteria (Figure S2). We deleted the phosphate sensing PAS domain [14] from PhoR and made fusions at both the conserved DHP domain (Dimerization and Histidine Phosphotransfer) and the charged region (CR). For the DHP region, fusions are at position 267 in Trg and link PhoR at successive one amino acid points, to account for helix rotation in the HK dimers. Most fusions have a basal signal in the absence of the ligand or no induction. DHP8, which fuses the Trg HAMP domain to position M197of PhoR (Figure S2A), showed the best ligand-dependent induction and was chosen for further analysis.

### Forming a complete plant synthetic signal transduction pathway with bacterial components and the rationally designed Trg-PhoR

**Figure 2:**
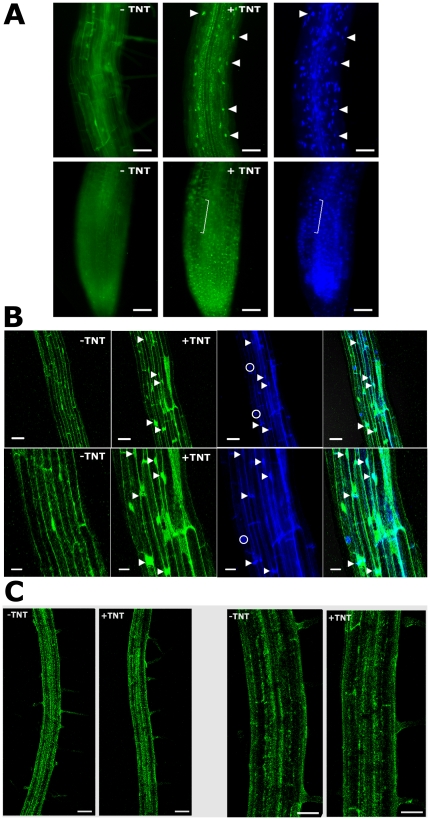
Signal-dependent nuclear translocation: Input from TNT induces nuclear translocation of PhoB-GFP. (*A*) Epi-fluorescence images of transgenic Arabidopsis roots containing ssTNT→Fls-Trg-PhoR→PhoB-GFP. The same root is shown before and after addition of the TNT ligand. *Top Panels*, Upper portion of a root. *Bottom panels*, Lower portion of a root. GFP fluorescence, bracket shows nuclei evident after TNT treatment. Right-most panels show DAPI nuclear staining. Arrowheads indicate nuclei. Scale bar  = 25 µm. (*B*) Confocal images of transgenic Arabidopsis roots containing ssTNT→Fls-Trg-PhoR→PhoB-GFP. *Top Panels*, Upper portion of a root, Scale bar  = 50 µm. *Bottom Panels*, Zoomed-in image of same root. Scale bar  = 20 µm. Panels with blue color show DAPI nuclear staining. Rightmost panels show overlay of GFP and DAPI images. Arrowheads indicate nuclei and circles indicate the base of a root hair. (*C*) Absence of signal dependent nuclear translocation of a mutated PhoB-GFP in the synthetic signal transduction pathway. Confocal images of transgenic Arabidopsis roots containing TNT receptor → Fls-Trg-PhoR → PhoB^D53A^-GFP. GFP fluorescence was diffuse both before and after treatment with the ligand, indicating no obvious nuclear translocation. From the left; first two panels, scale bar  = 100 µm; last two panels, scale bar  = 50 µm.

Bacterial signal transduction systems are capable of transmitting information from the exterior to a response using as few as two proteins whereas eukaryotic systems typically use multiple components. We tested if our bacterial derived components could be assembled for plant function by adapting each component with eukaryotic targeting sequences. We targeted the computationally re-designed receptor for TNT, TNT.R3 [5] to the apoplast, as described above for RBP, producing ss-TNT.R3. We re-engineered the DHP8 Trg-PhoR fusion for plant expression by adding an N-terminal signal peptide from a protein with known cell membrane localization (FLS2 [16]) to produce Fls-Trg-PhoR. We linked input from ss-TNT.R3 through the transmembrane Fls-Trg-PhoR to a bacterial response regulator PhoB (Figure 1). We previously detailed that PhoB is capable of translocation to the plant nucleus in response to HK activation (by exogenous cytokinin) in a signal-dependent and tissue-independent manner [12]. To initially test if the synthetic HK functioned in plants, we fused PhoB to GFP and determined if PhoB-GFP translocated to the plant nucleus in response to exogenous TNT. Transgenic plants containing ssTNT.R3→Fls-Trg-PhoR→PhoB-GFP were treated with the TNT ligand. Figure 2A-B shows that PhoB-GFP translocates to the nucleus in response to the ligand. Plants containing the same gene circuit but with the phospho-accepting Asp53 mutated (PhoB^D53A^-GFP) did not show ligand-dependent nuclear translocation (Figure 2C), indicating that the phospho-relay is required for ligand-mediated nuclear translocation of PhoB *in planta*.

PhoB has a well characterized DNA binding domain whose affinity for DNA is strongly enhanced by phosphorylation [17], [18], [19]. We used this DNA binding domain to design a synthetic promoter (*PlantPho*) and modified PhoB with a eukaryotic transcriptional activator, VP64 [12]. We showed that PhoB-VP64 could activate the PlantPho promoter fused to GUS in response to activation of endogenous HKs with exogenous cytokinin. To determine if input from the PBP receptor could transcriptionally activate our synthetic signal transduction system, we produced plants containing: ssTNT.R3 → Fls-Trg-PhoR → PhoB-VP64 → *PlantPho* promoter::GUS (Figure 1), hereafter called the complete signal transduction system. Approximately 80 primary Arabidopsis transformants were screened for ligand-induced GUS expression (Figure S3). Plant transformants typically showed ligand dependent induction. A few lines (e.g., number 66) showed repression; perhaps due to over-expression in a heterologous system.

**Figure 3:**
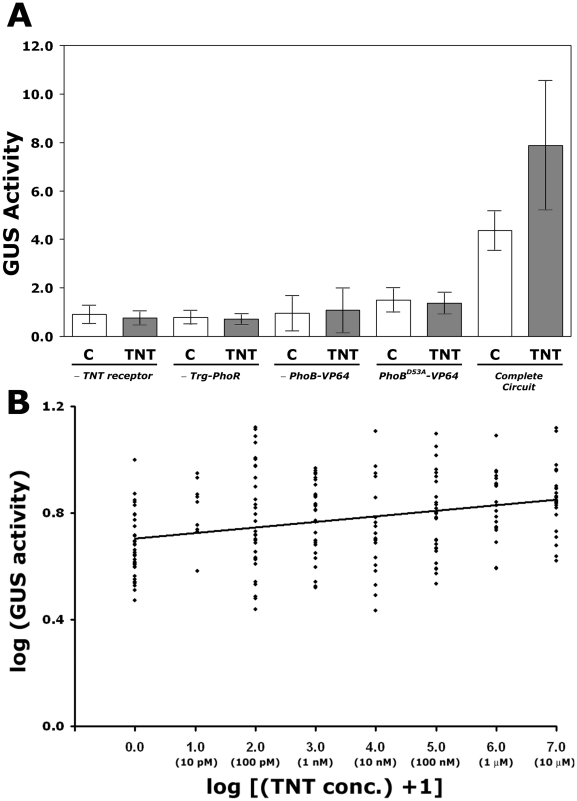
Ligand dependent induction of GUS via a synthetic signal transduction system in *Arabidopsis*. (*A*) GUS activity with and without 10 µm of the TNT ligand in paired leaves from transgenic plants serving as negative controls and transgenic plants containing the complete signal transduction system. Control (water), white column; TNT ligand, grey column. Transgenic lines, from left to right, are paired sets from lines containing all components but lacking the TNT receptor (-TNT receptor); lacking the chimeric histidine kinase (-Trg-PhoR); lacking the protein for nuclear translocation/transcriptional activation (-PhoB-VP64); with the phospho-relay disrupted (PhoB^D53A^-VP64). The complete circuit (right most columns) shows data from t-tests (one-tailed paired *t*-test; n = 29; mean GUS activity with ligand  = 7.88 (std. dev. = 2.66); mean GUS activity control = 4.33 (std. dev. = 0.79); t-ratio = 2.90; P = 0.0036). GUS activity expressed in nanomoles 4-MU.mg^−1^ protein.h^−1^. (*B*) GUS induction in transgenic plants in response to increasing ligand concentration. Linear regression of GUS activity as a function of ligand (TNT) concentration (n = 214; *F* = 21.12; *P* = 3.9×10^−93^; R^2^ = 0.09). Homozygous plants expressing the synthetic TNT sensing circuit (TNT receptor → Fls-Trg-PhoR → PhoB-VP64 → PlantPho promoter::GUS) were incubated with indicated amounts of the TNT ligand. For the log calculation, ligand concentrations are expressed in pM. GUS activity is as described in (*A*).

Figure 3A shows results of four control experiments, consisting of transgenic plants that lack one component of the complete signal transduction system (lack the receptor, lack the transmembrane HK, or lack the modified response regulator) or in which the critical phospho-accepting Asp53 residue was mutated. In these plants, there is no significant difference in the GUS activity with or without the TNT ligand, indicating that the complete signal transduction system and phospho-relay through PhoB-VP64 is required for transcriptional activation. In contrast, transgenic plants containing the complete gene circuit show significant induction of GUS in the presence of the TNT ligand. The ligand-dependent GUS accumulation was found in all 15 independent transgenic lines examined. Figure 3B shows a rigorous log-log plot of GUS activity in response to increasing amounts of the TNT ligand (10 pM to 10 µM). Arabidopsis transformants have a significant increase (*P* = 3.9×10^−93^) in GUS activity in response to the TNT ligand. Statistically, the low R^2^ indicates noise in the system, also apparent as GUS activity without the ligand and stochastic fluctuation. This variability may be a result of cross-talk with endogenous HK components [20], and/or accumulation of GUS in the two-week old plants tested. However, are results are highly reproducible, i.e., found in 15 independent heterozygous transgenic lines and the two independent lines taken to homozygosity. Collectively, these results indicate that our synthetic sensing gene circuit shows ligand-dependent gene expression in plants.

### Input from computationally re-designed receptors linked via synthetic signal transduction to a visible plant response

Ligand-dependent induction of our synthetic signal transduction system has stochastic fluctuations or noise. Excessive noise in biological system responses can be addressed by introducing a threshold to the engineered circuit that must be reached before activation occurs [21], [22]. Our previously described synthetic de-greening gene circuit shows a threshold-like response through generation of reactive oxygen species and photosystem disruption [7]. Hence, we linked output of our complete synthetic sensing gene circuit to the de-greening gene circuit by placing its genes (diRNA *POR*, protochlorophyllide oxidoreductase; *AtChlase*, chlorophyllase; *AtRCCR*, red chlorophyll catabolite reductase) [7] under control of the PlantPho promoter (Figure 1). The sensing gene circuit (Basta^R^, ssTNT.R3 → Fls-Trg-PhoR → PhoB-VP64) and de-greening gene circuit (Kan^R^, *PlantPho::de-greening* genes) were introduced into Arabidopsis and tobacco plants and primary transformants generated. We screened a total of 290 primary Arabidopsis transformants and 97 primary tobacco transformants for a ligand dependent response (Table S1) by following changes both visually and quantitatively. Our initial quantitative measurements followed changes in the maximum efficiency of photosystem II, expressed as *F_v_/F_m_* [23]. We previously documented that induction of the de-greening gene circuit produces a quantitative decrease in *F_v_/F_m_* [7]. Initial screens showed that detached leaves of plants containing the synthetic sensing and signal transduction system linked to the de-greening circuit respond to the ligand (as low as 10 pM TNT or 2.3 ppt, parts per trillion). Our initial screens of 290 independent transgenic Arabidopsis plants for response to 10 pM TNT produced approximately 20% responders (Table S1). As this screen with the very low level of the ligand may have missed some responders, we increased the ligand to 10 µM TNT in our screening of 97 independent primary transgenic tobacco plants. We found approximately 69% of the transgenic tobacco plants responded to the ligand (Table S1), typical for transgenic plants requiring expression of two T-DNAs.

Plants responding positively to the ligand were allowed to set seed and progeny were subjected to molecular analysis of induction and tested for heritability, visual responses, and quantitative responses. We first verified that the sensing gene circuit, assembled from bacteria genes, was fully expressed in later plant generations. Figure S4 shows expression analysis of all components of the sensing gene circuit (ss-TNT.R3, Fls-Trg-PhoR, PhoB-VP64) in second generation transgenic tobacco (NT4) and Arabidopsis (AT1) plants, although in Arabidopsis receptor levels (ssTNT.R3) are reduced.

**Figure 4:**
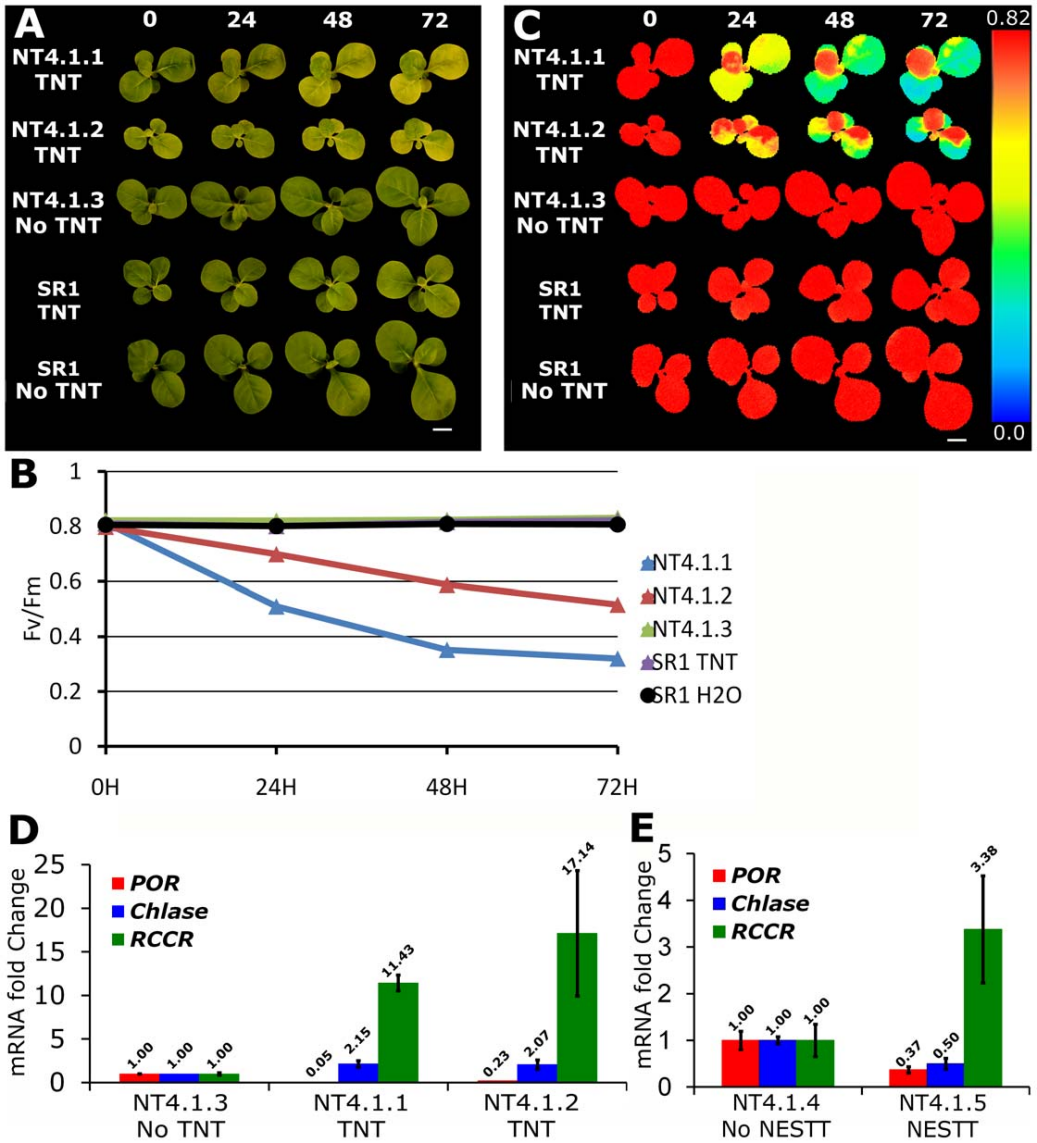
Response of transgenic plants to the TNT ligand. The roots of transgenic tobacco plants were exposed to 100 nM (23 ppb) by transferring plants to medium with TNT and preventing the leaves from contacting the TNT medium. (*A*) Visual response. Scale bar  = 1 cm. (*B*) Changes in maximum efficiency of photosystem II expressed as quantitative *F_v_/F_m_* values shown graphically or (*C*) Spatially. Scale bar  = 1 cm. (*D*) Real-time qRT-PCR confirming that plants responding as shown in *A, B*, and *C* results from activation of the de-greening gene circuit. *NT4.1.1TNT* and *NT4.1.2 TNT*, transgenic tobacco plants transferred to medium containing TNT; *NT4.1.3 No TNT*, transgenic plant transferred to medium without TNT; *SR1*, wild-type tobacco plants transferred and exposed to TNT; *SR1 No TNT*, wild-type tobacco plants transferred to medium without TNT. (*E*) Plants exposed to TNT vapor were analysed for induction of the de-greening circuit genes with Real-time qRT-PCR. *NT4.1.4 No NESTT*, transgenic tobacco plant from control tank; NT4.1.5 *NESTT*, transgenic tobacco plant exposed to TNT-NESTT. For *D* and *E*, *y*-axes indicate fold change in mRNA levels. Error bars equal one standard deviation. NT4.1.x represents the best responding transgenic line. Results with other lines are presented in Figure S6, with all results tabulated in Table S1.

We then tested the response of transgenic plants with the sensing and de-greening gene circuits to the TNT ligand using conditions that mimic real-world situations. One real world situation where a detector plant could function is sensing ligands beneath the surface, for example to detect landmines or pollutants. Transgenic tobacco plants were exposed to the TNT ligand in their roots and examined for a ligand response in the shoots. Figure 4 shows three distinct determinations for plant response to the ligand: visual, quantitative changes to the maximum efficiency of photosystem II (*F_v_/F_m_*) and with quantitative RT-PCR. *F_v_/F_m_* allowed remote quantification and we expressed the quantitative values spatially, where the numerical values are false-colored at the level of an individual pixel, and graphically, by averaging spatial values for the whole plant. Transgenic plants respond to 100 nM (23 ppb, parts per billion) TNT by producing a visual response within 24–48 hours (Figure 4*A*). Quantitative changes in *F_v_/F_m_* could easily be detected by 24 hours (Figure 4*B–C*) with the best expressing line, suggesting a response prior to this time point. Indeed, we previously detected the de-greening response at two hours [7]. Other lines showed a response within 48 hours (Figure S6, Table S1). In wild-type plants (SR1), the TNT ligand did not produce any visual or quantitative (*F_v_/F_m_*) responses (Figure 4*A*–*C*). Likewise, no effect was observed as a result of transplanting plants (NT 4.1.3, No TNT; SR1, No TNT). Changes induced with the de-greening gene circuit can also be followed with hyperspectral imaging, which allows remote sensing and application of automated recognition by computers [24].

To confirm that the visual and quantitative responses are a consequence of ligand dependent induction of the de-greening gene circuit we used quantitative RT-PCR. Figure 4D shows transcript levels for the endogenous *NtPOR* gene and the introduced AtChlase and AtRCCR genes in the transgenic control plant (NT 4.1.3) and transgenic plants exposed to TNT. Note that wildtype SR1 plants do not contain introduced genes precluding comparison with qRT-PCR. Plants exposed to TNT showed transcript changes predicted for induction of the de-greening circuit: *NtPOR* levels declined to 5% and 23% of controls, *AtChlase* levels increased (two-fold), and *AtRCCR* levels increased (11- and 17-fold). These results indicate that the TNT ligand causes induction of the de-greening gene circuit and the response is not caused by stress, as expression of *POR* is known to increase in response to stress [7], [25], [26]. The expression levels of *AtRCCR* and *AtChlase* from the same PlantPho promoter differ, possibly from differences in mRNA stability for *AtRCCR* and *AtChlase*.

In bacteria the computationally re-designed receptors show a high degree of specificity, with related ligands showing 2–3 fold less affinity [5]. We assayed whether this specificity is retained in our transgenic plants using the TNT breakdown products 2,4- and 2,6-dinitrotoluene previously tested in bacteria [5]. The transgenic plants responded weakly to the breakdown products, comparable to the bacterial response (Figure S5), suggesting the specificity seen in bacteria is retained in plants.

Another real world application of a detector plant is to sense substances in the air (e.g., explosives, pollutants). Because rigorously providing known explosive vapor at a given concentration is a technically intensive and expensive approach, we exposed our transgenic plants to vapor provided by TNT-NESTT (Non-hazardous Explosives for Security Training and Testing) [27]. While initial reports indicated that NESTT provides TNT vapor at approximately 8.6 ppb at 25°C [27], more recent analysis indicates the NESTT volatiles are largely a mixture formed of the TNT breakdown products 2,4- and 2,6-dinitrotoluene, with only a minor portion being TNT [28]. NESTT vapor induces minimal visual changes in the transgenic plants that are comparable to, or slightly stronger than those observed in the presence of the 2,4- and 2,6-dinitrotoluene products. Quantitative RT-PCR analysis of the de-greening circuit genes (Figure 4*E*) corroborates the weak response observed in the transgenic plants, with endogenous *NtPOR* mRNA levels decreasing to approximately one-third of the control levels, and *AtRCCR* increasing three-fold. However, *AtChlase* mRNA levels in the transgenic plants, which were expected to increase, were reduced to half the levels observed in control plants (Figure 4*E*) (see discussion).

## Discussion

There is a pressing need for simple and cost effective means to monitor environments for pollutants, explosives and chemical agents. We describe the production of transgenic plants using input from computationally re-designed receptors that signals through a synthetic signal transduction system. We linked the input to a threshold-like response that produces visual and quantitative changes and also allow remote sensing and application of automated recognition by computers [24]. Although our results show that it is possible to produce highly sensitive prototype detector plants, technical issues prevent these plants from being used in the real world at this time. Further refinement of our system could lead to a simple and inexpensive means to detect pollutants, explosives and terrorist agents.

Evolutionarily conserved HK components are used by plants and bacteria to sense and respond to their environments. Our discovery that this conservation also extends to the level of HK transmembrane signaling allowed us to design a test system, whereby transmembrane fusions can be rapidly developed and tested in bacteria and then, with the addition of proper targeting sequences, function in plants. One limitation of this first generation plant system is stochastic fluctuation of the response (Figure 3*B* and Figure S3). While this variability likely has multiple causes, we found that GUS is expressed without the ligand and the variation exists over the range of ligand concentrations tested (Figure 3*B*). GUS stability likely makes some contribution to the noise [29]. In addition, because we developed PhoB-VP64 for plants by activating endogenous HKs with exogenous cytokinins [12], it is likely that our synthetic signal transduction system (receptor → HK → PhoB:VP64 → signal receptive promoter) has false input or cross-talk from plant HK components. Plant HK components are involved in hormonal and possibly light-mediated signal transduction processes, hence it is difficult to eliminate their input in our system. The abilities we developed here, to rationally design and test HK components in bacteria followed by functionality in plants, allow us to apply directed evolution [30] and high throughput methods that may allow later generations to operate without input from plant HK components.

In our system, plants sense and then respond to pM-µM levels of the extracellular ligand. We measured the ligand induced response using four different methods: quantitative measurements of GUS, visual changes, quantitative changes in photosystem II efficiency (*F_v_/F_m_*), and with quantitative RT-PCR. GUS induction is found with pM levels of the TNT ligand whereas the de-greening circuit response is typically found with low nM levels. Because the response from the de-greening gene circuit involves threshold-like behavior, accuracy at lower levels of the ligand is currently more difficult to follow. The line displaying the best response, NT4, contains multiple T-DNAs (Table S2) with segregation of the de-greening circuit genes (Kan^R^) suggesting linkage. Lines with one copy of each gene circuit consistently respond to TNT (Figure S6), though the response is not as strong. While the threshold-like behavior of the de-greening circuit likely contributes to these differences, we cannot rule out the need to balance our synthetic HK signal transduction components for optimal transmission, in a manner similar to that in bacteria [31]. These data suggest that it should be possible to design improvements to the current non-optimized components. Approaches successful in other systems, such as modeling signal transduction and synthetic biology methodologies [32] could improve future generations of detector plants by enhancing signal transmission and providing means to deal with biological “noise”. Likewise, the ability to tune gene circuits [33], [34], [35] may allow our detector plant system to be adjusted as applications demand (e.g., rapid response for transportation hubs or better reset for environmental monitoring).

We used quantitative RT-PCR to confirm and measure activation of the de-greening gene circuit (Figure 4*D–E*). In tobacco, we measured a reduction in the endogenous *POR* genes (*NtPOR*), and induction of the *AtRCCR* and *AtChlase* transgenes in response to TNT. In our vapor experiments, *NtPOR* and *AtRCCR* responded as predicted, but with a reduced level, perhaps because NESTT primarily supplies the significantly less responsive analogs (2,4- and 2,6-dinitrotoluene). However, *AtChlase* levels did not increase, but were found to decrease. This response defies a simple explanation.

Our system's current parameters strongly suggest it possesses wide utility. In the broader sense, our work describes a modular, biological input-output system. As such, it can be used to control a visual response, described here, or a trait of interest (e.g., flowering time, biofuel trait). From the basic research perspective, the ability to generate chimeric histidine kinase molecules could allow the dissection of specific functions of the individual components in multigene families. From the applied perspective, our detection level is well within or better than that needed for real world use. For example dogs, typically employed to detect explosives and drugs, have somewhat variable abilities, but in general their detection range is thought be in the tens of ppb to 500 ppt [36], [37]. As such our detector plants are approximately 100-fold better (GUS data) or equivalent (de-greening response) to the detection abilities of dogs. Environmental pollutants are a concern at levels that vary with the given pollutant and environment. However, a detection system in the pM-nM range would find utility for most environmental pollutants.

The computationally re-designed receptors provide a modular platform that could theoretically allow design and use of highly specific receptors for most small molecules. Recently, an *in vitro* analysis of some computationally re-designed receptors found properties inconsistent with published binding characteristics [38]. While our analysis does not provide any details about how the computationally re-designed receptor binds the TNT ligand *in planta*, we consistently see reproducible signal transduction in our *in vivo* systems (bacterial and plant). One possibility is that previously-described receptor instability *in vitro* [5] precludes typical biochemical analysis, whereas in bacteria or plants, the receptor is continuously produced, and hence activates signal transduction. While we found an increasing trend with increasing TNT concentrations (Figure 3*B*), our system does not have the predictability reported by Looger et al [5]. However, as computational re-design further improves, a greater variety of receptors recognizing a wider range of molecules will be available as possible inputs.

Our transgenic plants are one of the first examples of detector plants and a fully synthetic signaling pathway in a higher eukaryotic organism. Our synthetic signal transduction system was built using a modular assembly of bacterial and plant protein domains. With refinement of signal transduction components, stronger and more specific signaling pathways may be possible. Also, by fine tuning expression levels with feedback regulation [34], digital like regulation can be added that enhances sensitivity and provides biological memory. The extraordinary adaptive potential of this system makes it likely that improved versions are soon to follow.

## Materials and Methods

### Plant material

Transformation of Arabidopsis (ecotype Col-0) and *Nicotiana tabacum* (Petit Havana SR-1) was accomplished with Agrobacterium-mediated methods.

### Plasmid constructs

PhoR, Trg, and EnvZ genes or gene fragments were amplified by PCR from *E. coli.* A more detailed description is provided in Materials and Methods S1.

### Production of plants with detection and response gene circuits

The Pex secretory sequence (ss) [39] was fused to the TNT.R3 receptor [5] using overlapping extension PCR and the resulting ssTNT.R3 gene cloned into pCB302-3. The signal peptide from the Arabidopsis *FLS2* gene (At5g46330) (16) was fused to the start codon of Trg-PhoR, cloned into a pBS-PNOS-TNOS-TB intermediate vector, and sub-cloned into pCB302-3. The complete synthetic signaling circuit was obtained by co-transforming the above-described plasmid containing ssTNT.R3 and Fls-Trg-PhoR with a previously described plasmid containing the PlantPho system [12] which includes the signal PhoB-VP64 under control of an FMV promoter and a PlantPho::GUS-TNOS reporter. The de-greening circuit was produced by replacing the 10xN1 promoter driving *POR* diRNA, *Chlorophyllase*, and *Rccr* from circuit number one [7] with the PlantPho promoter [12]. Additional details are provided in Materials and Methods S1.

### Statistical Analysis

All statistical analyses were performed using JMP software, v. 6.0.3 (SAS Institute). All the assumptions of parametric statistics were tested and met.

## Supporting Information

Figure S1 Addition of a plant signal peptide targets computationally re-designed receptors to the plant apoplast. We replaced the bacterial periplasmic signal peptide from RBP with a plant signal peptide. Transient assays were done in onion epidermal cells using the plant signal sequence fused to RBP and a GFP reporter (ssRBP-GFP fusion protein) with RBP fused to GFP as a control. The signal sequence correctly targeted the bacterial periplasmic protein to the plant apoplast. A1, ssRBP-GFP localizes to the apoplast of plasmolyzed cells. A2, brightfield images of plasmolyzed cells. A3, overlay images of A1 and A2. B1, identical experiment demonstrating ssRBP-GFP localizes to the apoplast of plasmolyzed cells. B2, brightfield images of plasmolyzed cells. B3, overlay images of B1 and B2. C1, RBP-GFP localized to the cytoplasm of plasmolyzed cells. C2, brightfield images of plasmolyzed cells. C3, overlay images of C1 and C2. White arrows indicate cell walls, red arrows indicate protoplast.
(TIF)

Figure S2 **Bacterial testing of transmembrane fusions and diagrams of proteins** (*A*) Numerous fusion points were tested at each domain junction in PhoR (*DHP, CR*) with the HAMP domain of Trg, as described in the chart. The fusion designated *DHP8* was re-engineered and used in plants. *x*-axis, Trg-PhoR fusion names, with precise fusion points indicated in the table (below). *y-*axis, GFP fluorescence intensity. *Apo*, control; *10 mM Ribose*, ligand present. (*B*) Diagram of the transmembrane proteins and a sample fusion. Trg's extracellular sensing domain is fused to HK PhoR after removing the PAS domain from PhoR. *HAMP*, *DHP* and *hATPase* refer to functional domains of Trg-PhoR (11).
(TIF)

Figure S3 Transcriptional Activation: TNT-dependent changes in GUS expression in paired leaves from ten independent primary transgenic plants containing ssTNT→Fls-Trg-PhoR→PhoB-VP64→PlantPho::GUS. GUS activity expressed in nmoles 4-MU.mg^−1^ protein.h^−1^.
(TIF)

Figure S4 Reverse transcriptase-polymerase chain reaction (RT-PCT) analysis of synthetic sensing and signaling components confirms expression of components of the sensing gene circuit. Synthetic sensing components: ssTNT, *ssTNT receptor;* TrgPhoR, *Fls-Trg-PhoR;* PhoB, *PhoB-VP64*. Control transcript genes: Tub, *N. tabacum α- tubulin*; Cyc, Arabidopsis *cyclophlin*. Samples: SR1, *Nicotiana tabacum* wildtype control*;* NT4, *tobacco line 4.1.1*(response to TNT shown in Figure 4); Col, Arabidopsis ecotype Columbia*;* AT1, second generation Arabidopsis line AT1.1.
(TIF)

Figure S5 Test for ligand specificity in transgenic tobacco plants. Plants from the same generation used in TNT assays (Fig. 4) were used to test the response to TNT analogs, 2,4- and 2,6-dinitrotoluene with an identical setup. (*A*) Response of transgenic plants (NT4.1.10-NT 4.1.12) to 100 nM 2,4-dinitrotoluene (2,4-DNT) and (*B*) 100 nM 2,6-dinitrotoluene (2,6-DNT)(NT 4.1.13-NT4.1.15). While no visual response was evident, a weak response was measured in *F_v_/F_m_*. Wild-type (*SR1*) plants were also exposed to each analog and no response was detected. Scale bar  =  1 cm.
(TIF)

Figure S6 Response of transgenic tobacco plants with one copy of each gene circuit, de-greening gene circuit and synthetic sensing gene circuit. Transgenic plant lines with one copy of each gene circuit were exposed to 100 nM TNT in a setup identical to that of NT4. Like NT4, these lines produce a consistent response. However the response is less than that seen with NT4. Scale Bar  =  1 cm.
(TIF)

Table S1 Number of primary transgenic tobacco and Arabidopsis lines generated and their initial response as scored in a leaf assay. The response ranged "Strong", leaves with an obvious visual response and significant reduction in *F_v_/F_m_* (generally less than a value of 0.5) to "Slight", leaves with an equivocal visual response and small reduction in *F_v_/F_m_*. Arabidopsis lines were not scored as “moderate” (NS).
(DOC)

Table S2 Observed segregation of T-DNA's in transgenic tobacco lines. The sensing gene circuit is contained on a T-DNA providing resistance to Basta whereas the de-greening gene circuit is on a T-DNA providing resistance to kanamycin. Genetic analysis indicates NT4 has multiple T-DNAs whereas NT9 has one T-DNA for each introduced trait. The multiple T-DNAs of NT4 segregated together suggestion some type of linkage. In the third generation, NT4.1, shows significantly less Basta resistant plants that predicted, consistent with gene silencing. *Km*, kanamycin. *R*, resistant. *S*, sensitive.
(DOC)

Materials and Methods S1 Plant Material, Transgenic Plant Production and Growth Conditions.
(DOC)
