# Supplementary figures and images for: Programmable Ligand Detection System in Plants through a Synthetic Signal Transduction Pathway
Source: PLoS One. 2011 Jan 25;6(1):e16292. doi: 10.1371/journal.pone.0016292 (PMC3026823; doi:10.1371/journal.pone.0016292)

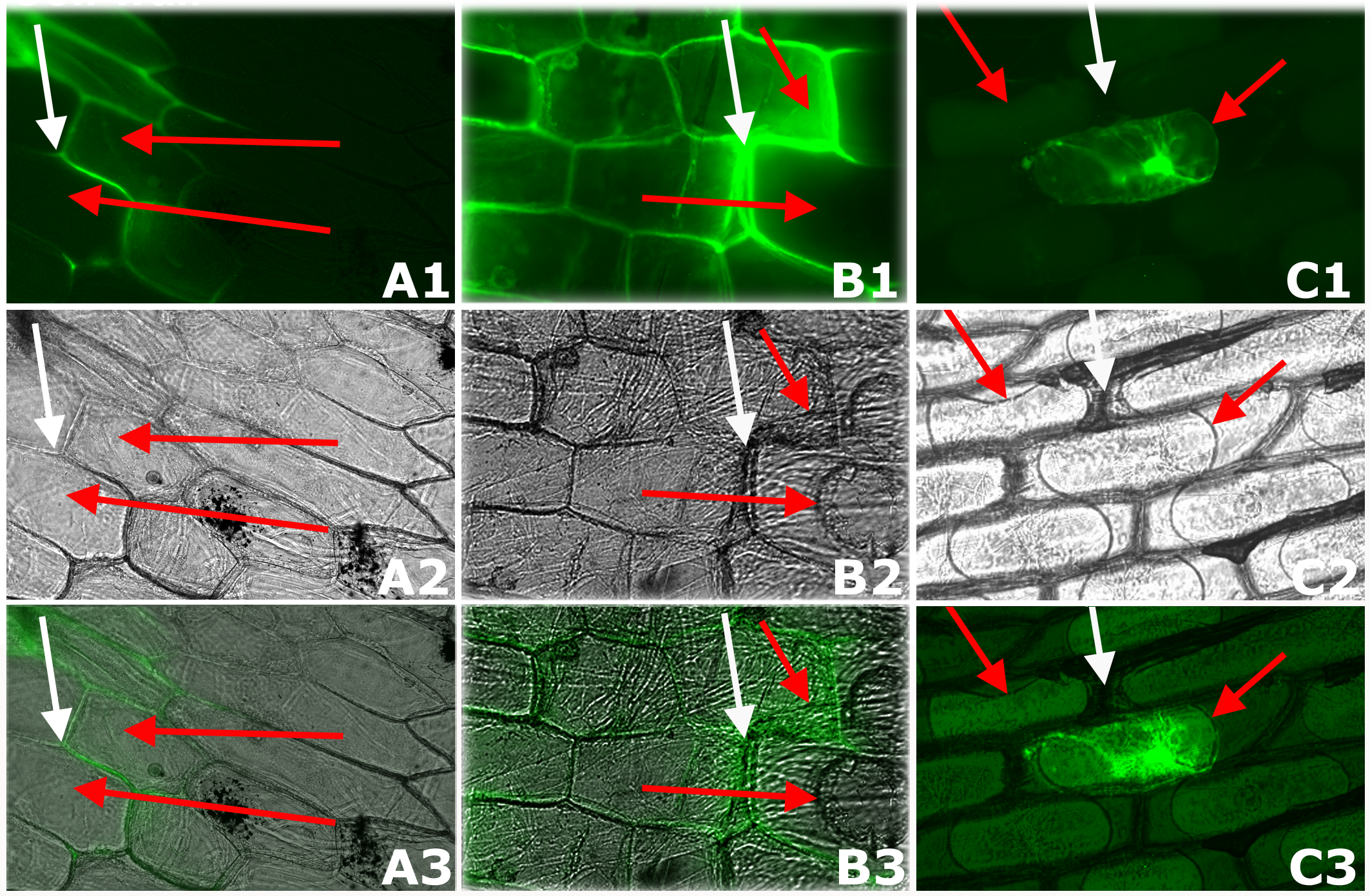

Supplement: Figure S1 — Addition of a plant signal peptide targets computationally re-designed receptors to the plant apoplast. We replaced the bacterial periplasmic signal peptide from RBP with a plant signal peptide. Transient assays were done in onion epidermal cells using the plant signal sequence fused to RBP and a GFP reporter (ssRBP-GFP fusion protein) with RBP fused to GFP as a control. The signal sequence correctly targeted the bacterial periplasmic protein to the plant apoplast. A1, ssRBP-GFP localizes to the apoplast of plasmolyzed cells. A2, brightfield images of plasmolyzed cells. A3, overlay images of A1 and A2. B1, identical experiment demonstrating ssRBP-GFP localizes to the apoplast of plasmolyzed cells. B2, brightfield images of plasmolyzed cells. B3, overlay images of B1 and B2. C1, RBP-GFP localized to the cytoplasm of plasmolyzed cells. C2, brightfield images of plasmolyzed cells. C3, overlay images of C1 and C2. White arrows indicate cell walls, red arrows indicate protoplast. (TIF) [file pone.0016292.s001.tif]

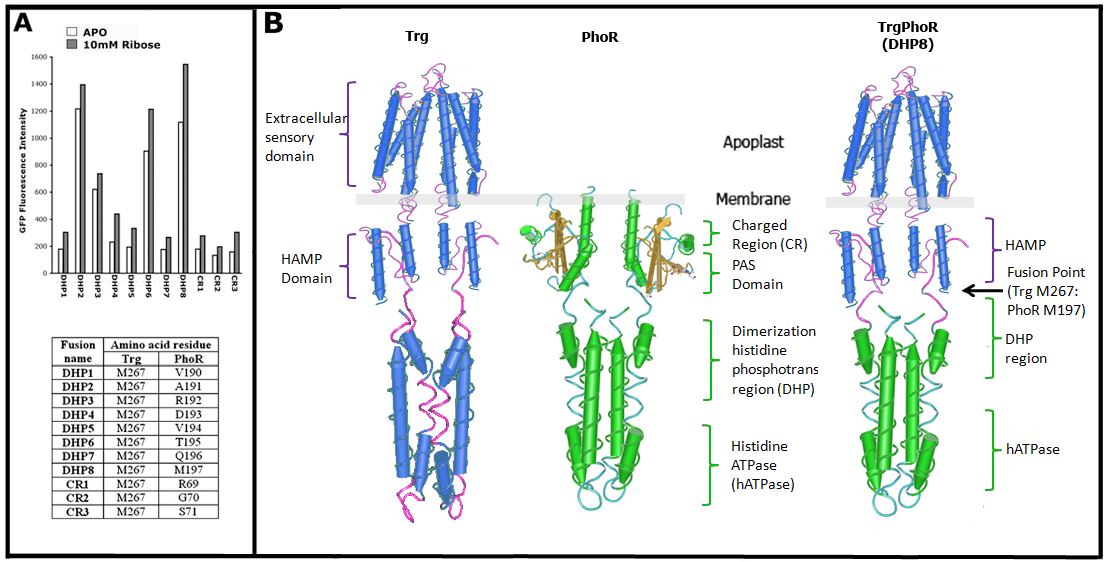

Supplement: Figure S2 — Bacterial testing of transmembrane fusions and diagrams of proteins (A) Numerous fusion points were tested at each domain junction in PhoR (DHP, CR) with the HAMP domain of Trg, as described in the chart. The fusion designated DHP8 was re-engineered and used in plants. x-axis, Trg-PhoR fusion names, with precise fusion points indicated in the table (below). y-axis, GFP fluorescence intensity. Apo, control; 10 mM Ribose, ligand present. (B) Diagram of the transmembrane proteins and a sample fusion. Trg's extracellular sensing domain is fused to HK PhoR after removing the PAS domain from PhoR. HAMP, DHP and hATPase refer to functional domains of Trg-PhoR (11). (TIF) [file pone.0016292.s002.tif]

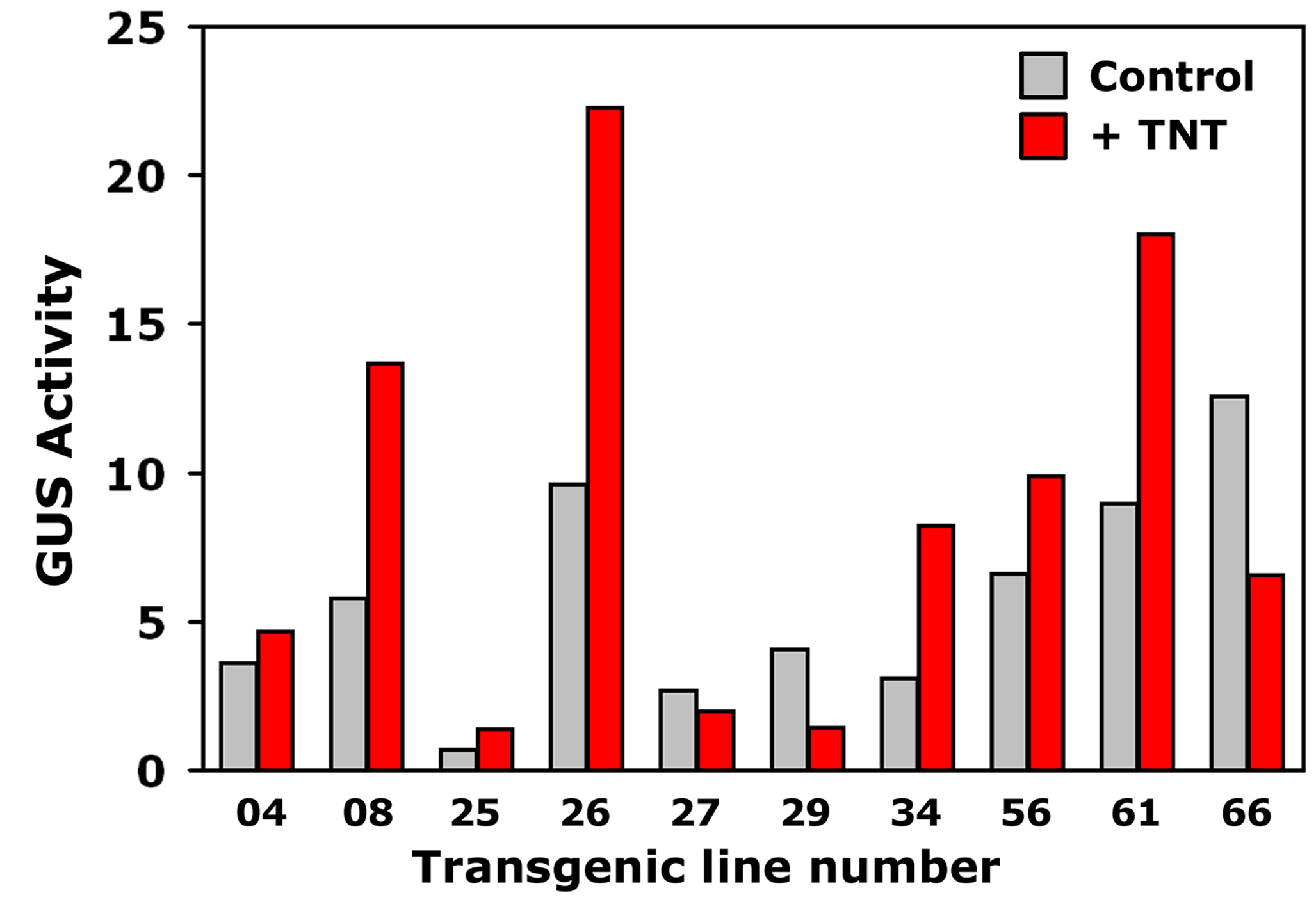

Supplement: Figure S3 — Transcriptional Activation: TNT-dependent changes in GUS expression in paired leaves from ten independent primary transgenic plants containing ssTNT→Fls-Trg-PhoR→PhoB-VP64→PlantPho::GUS. GUS activity expressed in nmoles 4-MU.mg−1 protein.h−1. (TIF) [file pone.0016292.s003.tif]

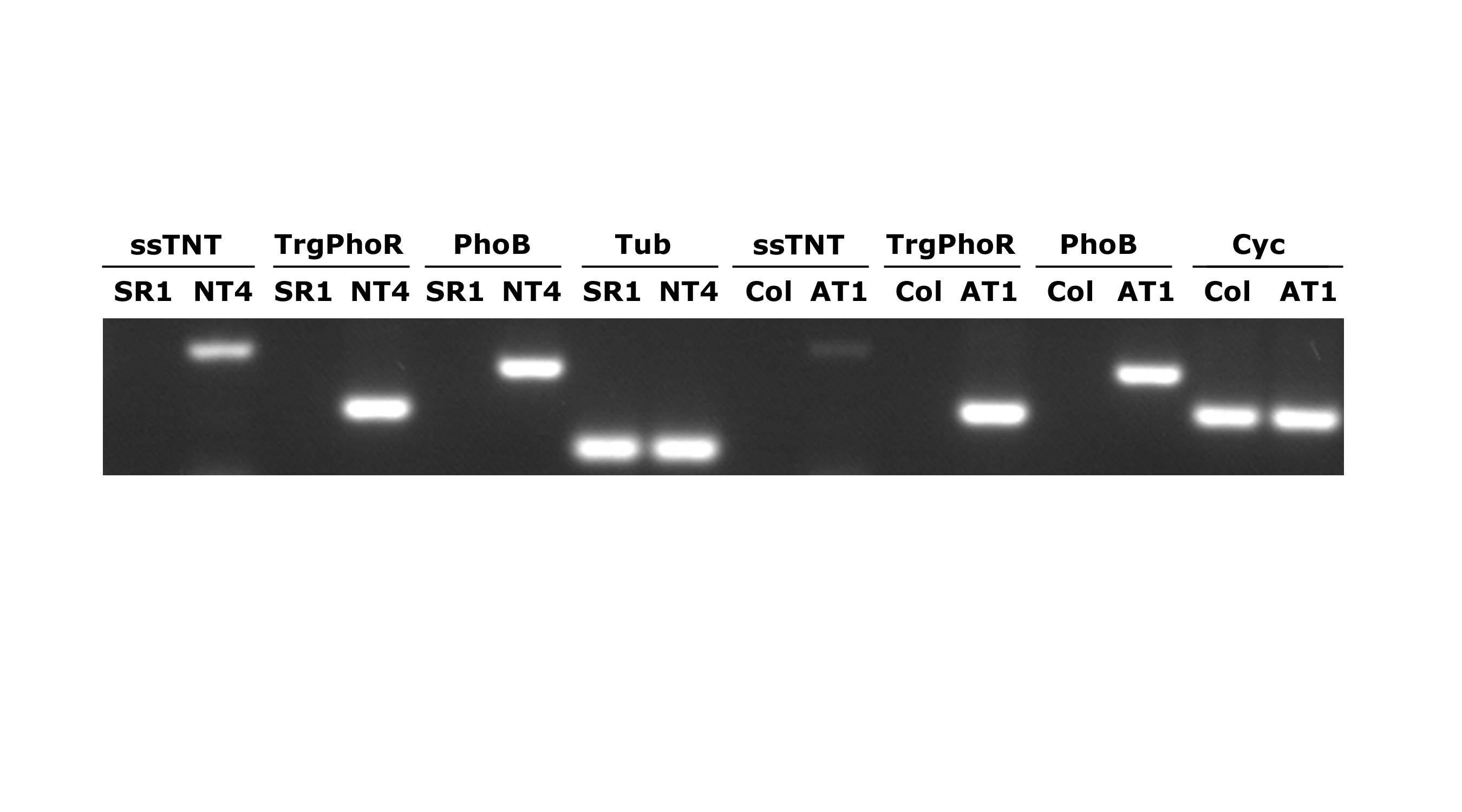

Supplement: Figure S4 — Reverse transcriptase-polymerase chain reaction (RT-PCT) analysis of synthetic sensing and signaling components confirms expression of components of the sensing gene circuit. Synthetic sensing components: ssTNT, ssTNT receptor; TrgPhoR, Fls-Trg-PhoR; PhoB, PhoB-VP64. Control transcript genes: Tub, N. tabacum α- tubulin; Cyc, Arabidopsis cyclophlin. Samples: SR1, Nicotiana tabacum wildtype control; NT4, tobacco line 4.1.1(response to TNT shown in Figure 4); Col, Arabidopsis ecotype Columbia; AT1, second generation Arabidopsis line AT1.1. (TIF) [file pone.0016292.s004.tif]

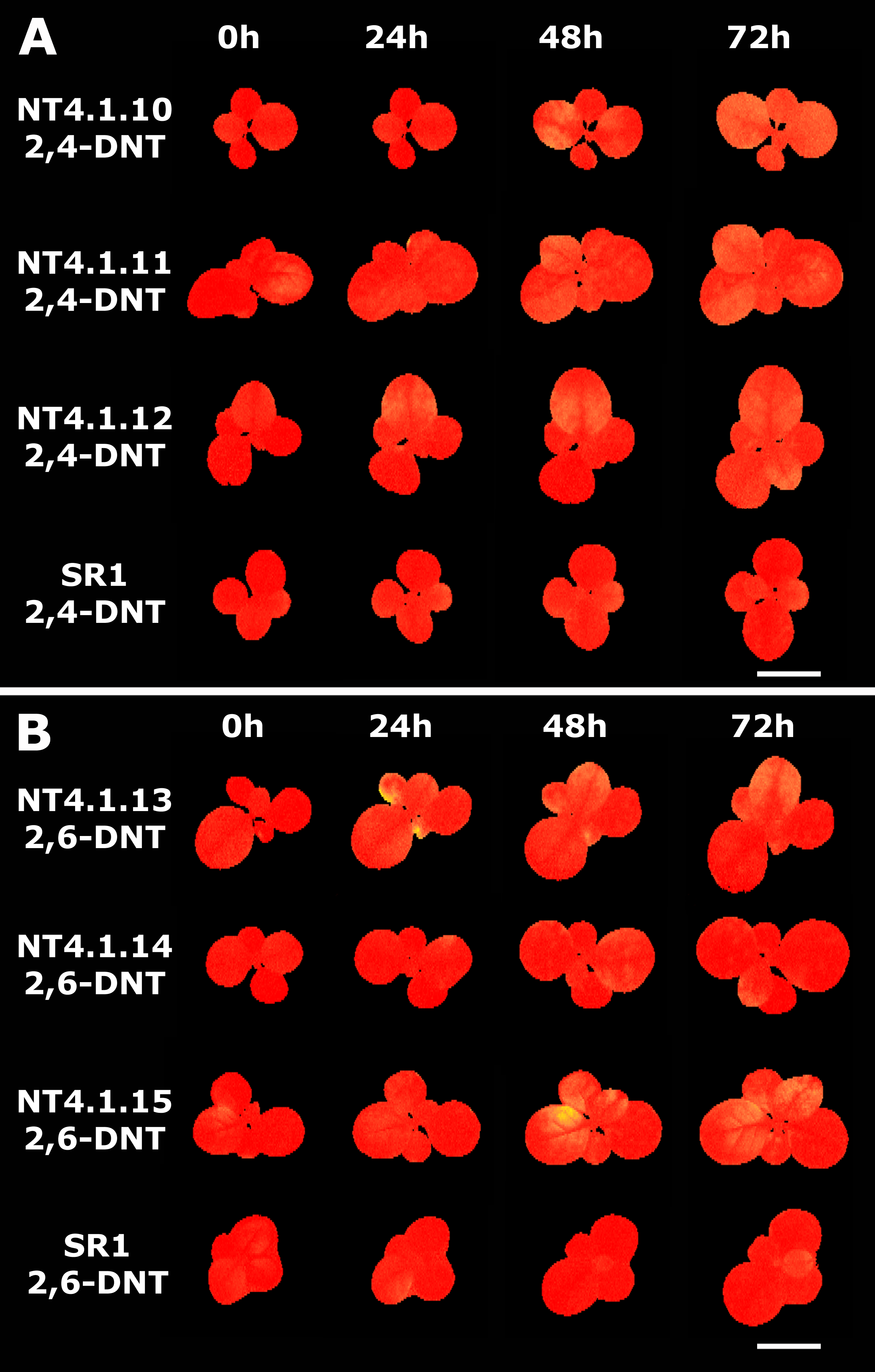

Supplement: Figure S5 — Test for ligand specificity in transgenic tobacco plants. Plants from the same generation used in TNT assays (Fig. 4) were used to test the response to TNT analogs, 2,4- and 2,6-dinitrotoluene with an identical setup. (A) Response of transgenic plants (NT4.1.10-NT 4.1.12) to 100 nM 2,4-dinitrotoluene (2,4-DNT) and (B) 100 nM 2,6-dinitrotoluene (2,6-DNT)(NT 4.1.13-NT4.1.15). While no visual response was evident, a weak response was measured in Fv/Fm. Wild-type (SR1) plants were also exposed to each analog and no response was detected. Scale bar = 1 cm. (TIF) [file pone.0016292.s005.tif]

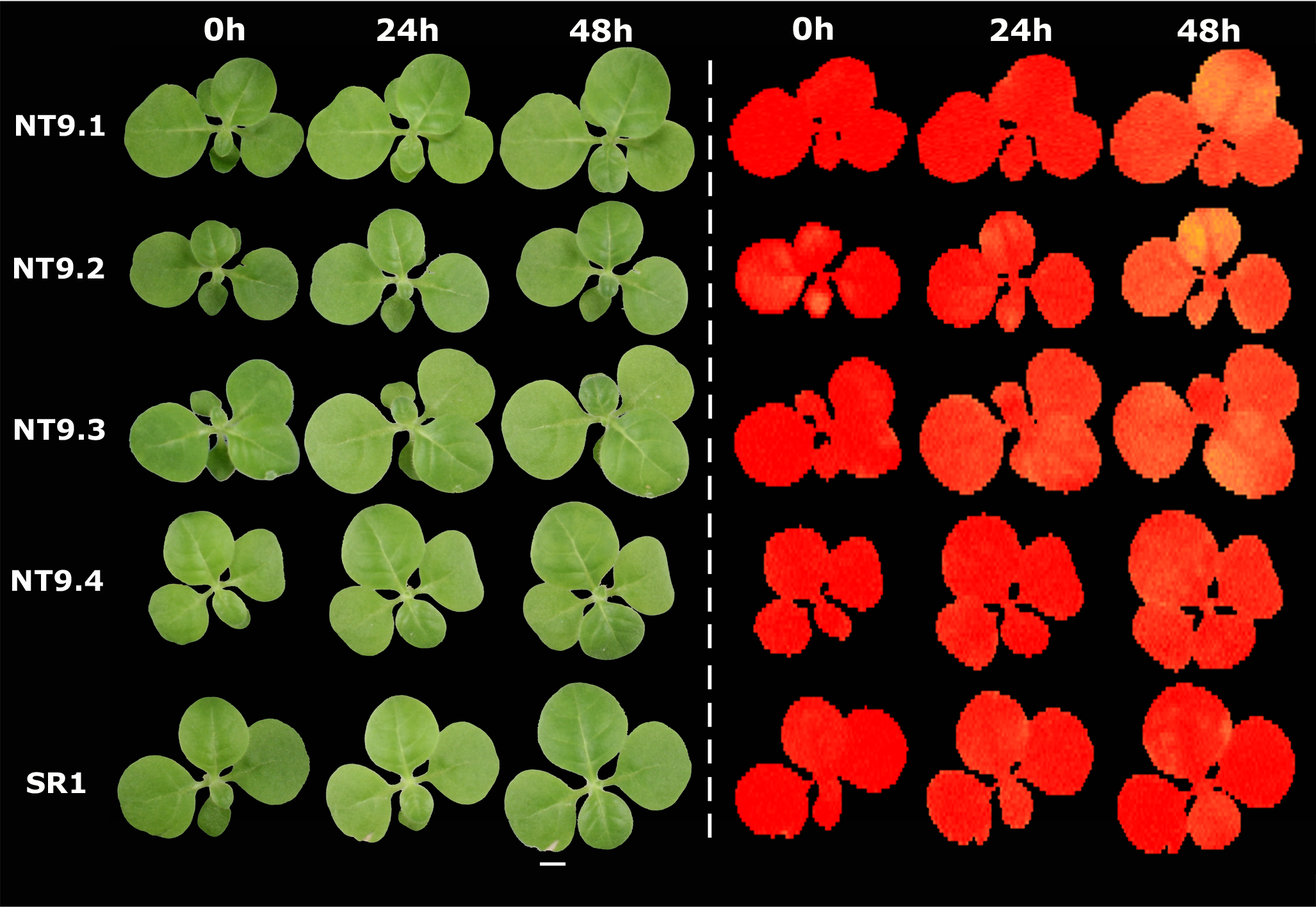

Supplement: Figure S6 — Response of transgenic tobacco plants with one copy of each gene circuit, de-greening gene circuit and synthetic sensing gene circuit. Transgenic plant lines with one copy of each gene circuit were exposed to 100 nM TNT in a setup identical to that of NT4. Like NT4, these lines produce a consistent response. However the response is less than that seen with NT4. Scale Bar = 1 cm. (TIF) [file pone.0016292.s006.tif]
