# Supplementary material for: Programmable Ligand Detection System in Plants through a Synthetic Signal Transduction Pathway
Source: PLoS One. 2011 Jan 25;6(1):e16292. doi: 10.1371/journal.pone.0016292 (PMC3026823; doi:10.1371/journal.pone.0016292)
Supplement: Table S1 — Number of primary transgenic tobacco and Arabidopsis lines generated and their initial response as scored in a leaf assay. The response ranged "Strong", leaves with an obvious visual response and significant reduction in Fv/Fm (generally less than a value of 0.5) to "Slight", leaves with an equivocal visual response and small reduction in Fv/Fm. Arabidopsis lines were not scored as “moderate” (NS). (DOC) [file pone.0016292.s007.doc]

| **Degreening Response** | **Number of Tobacco Responders** | **Number of Arabidopsis Responders** |
| --- | --- | --- |
| Strong | 6 | 4 |
| Good | 22 | 21 |
| Moderate | 22 | NS |
| Slight | 17 | 37 |
| No Response | 30 | 228 |
| **Total Assayed** | **97** | **290** |
