# Supplementary material for: Programmable Ligand Detection System in Plants through a Synthetic Signal Transduction Pathway
Source: PLoS One. 2011 Jan 25;6(1):e16292. doi: 10.1371/journal.pone.0016292 (PMC3026823; doi:10.1371/journal.pone.0016292)
Supplement: Table S2 — Observed segregation of T-DNA's in transgenic tobacco lines. The sensing gene circuit is contained on a T-DNA providing resistance to Basta whereas the de-greening gene circuit is on a T-DNA providing resistance to kanamycin. Genetic analysis indicates NT4 has multiple T-DNAs whereas NT9 has one T-DNA for each introduced trait. The multiple T-DNAs of NT4 segregated together suggestion some type of linkage. In the third generation, NT4.1, shows significantly less Basta resistant plants that predicted, consistent with gene silencing. Km, kanamycin. R, resistant. S, sensitive. (DOC) [file pone.0016292.s008.doc]

|  | **Observed Segregation of Progeny** | |  |  |  |
| --- | --- | --- | --- | --- | --- |
| **Line** | **Km^R^ Seedlings** | **Km^S^ Seedlings** | **Theoretical Segregation Ratio Tested** | χ^2^ | **P-Value** |
| NT9 | 36 | 8 | 3:1 | 1.09 | 0.296 |
| NT4 | 189 | 5 | 63:1 | 1.30 | 0.935 |
| NT4.1 | 96 | 0 | 63:1 | 1.52 | 0.940 |
|  |  |  |  |  |  |
|  | **Observed Segregation of Progeny** | |  |  |  |
| **Line** | **BASTA^R^ Seedlings** | **BASTA^S^ Seedlings** | **Theoretical Segregation Ratio Tested** | χ ^2^ | **P-Value** |
| NT9 | 33 | 13 | 3:1 | 0.26 | 0.6 |
| NT4 | 52 | 23 | 3:1 | 1.28 | 0.258 |
| NT4.1 | 16 | 79 | 3:1 | 171.4 | <0.0001 |
