# Supplementary material for: Programmable Ligand Detection System in Plants through a Synthetic Signal Transduction Pathway
Source: PLoS One. 2011 Jan 25;6(1):e16292. doi: 10.1371/journal.pone.0016292 (PMC3026823; doi:10.1371/journal.pone.0016292)
Supplement: Materials and Methods S1 — Plant Material, Transgenic Plant Production and Growth Conditions. (DOC) [file pone.0016292.s009.doc]

**Supporting Information**

**Antunes et al.**

**SI Materials and Methods**

***Plant Material, Transgenic Plant Production and Growth Conditions.*** *Arabidopsis thaliana* (ecotype Col-0) and *Nicotiana tabacum* (Petit Havana SR-1) were used for transformation using standard *Agrobacterium*-mediated methods. Plasmids were assembled as described below and transferred into *Agrobacterium tumefaciens* strain GV3101 by electroporation. Arabidopsis plants were transformed by the floral dip method (1). Tobacco plants were transformed as previously described (2), except that whole leaves instead of leaf disks were used. Leaves were wounded with forceps prior to *Agrobacterium* inoculation. Transgenic T_0_ plants were selected on culture medium containing full-strength MS media (3), 0.8% agar, pH 5.7. Kanamycin (50 mg/L) (Sigma-Aldrich, St. Louis, MO), and/or glufosinate (5 mg/L) (Basta; Crescent Chemical, Islandia, NY) were added to the medium as needed for the selection required for the transgenic plants. T_0_ lines were selected and allowed to self-pollinate. Transgenic seeds were sterilized and cold treated to synchronize germination for 1-3 days at 4°C, and were grown at 23-25°C under 16 hours light (70-100 µE.m^-2^.s^-1^ fluorescent light)/ 8 hours dark cycle, in either a Percival AR75L growth chamber or light shelf.

***Plasmid Constructs.*** To assemble fusions for testing in bacteria, PhoR, PhoB, and Trg genes or gene fragments were amplified by PCR from *E. coli* (Cold Spring Harbor Collection strain ten genomic DNA). PhoR fusions were constructed using overlapping extension PCR (4) to join the appropriate fragments at desired junctions. Fusions were digested with *Nhe*I and *Hind*III, inserted into pACYC177, and driven by an inserted lacIq promoter. Fusions were produced, cloned, and verified in the same manner described for PhoR fusions. In order to test PhoR-mediated gene regulation in bacteria, the PhoA promoter was amplified by PCR and fused to a GFPuv gene (5) by overlapping extension PCR and then cloned into pBR322 using *Eco*RI and *Pst*I sites. Chemically competent RU1012 bacterial cells containing the PhoA::GFP reporter plasmid were transformed with PhoR fusion plasmids and plated on 100 µg/mL ampicillin and 20 µg/mL tetracycline. Liquid cultures were inoculated with bacteria containing both plasmids, grown to an OD_600_ of 0.4, diluted 10^-6^, and plated on 2x YT media (1 L: 16 g Bacto-Tryptone, 10 g Bacto-Yeast Extract, 5 g NaCl, 15 g Agar, pH 7.0 with NaOH) containing 100 µg/mL ampicillin, 20 µg/mL tetracycline, with or without 10 mM ribose as the ligand. Plates were grown at 37^o^C for 12 hours then at room temperature for another 24 hours. GFPuv response was quantified by re-suspending colonies from the plate, diluting total cell count to the same OD_600_, lysing bacteria with BugBuster Protein Extraction Reagent (Novagen, Madison, WI) for one hour at room temperature, pelleting debris by centrifugation for ten minutes at 16,000 rpm, then measuring GFPuv fluorescence of supernatant on a SLM Aminco-Bowman Series 2 fluorometer. GFPuv fluorescence was measured by exciting at 395 nm and recording the emission at 510 nm using a 2 nm slit width.

***Receptor Targeting to the Apoplast.*** For transient assays of protein localization in plants, the secretory sequence from a Pex gene (pollen extensin-like protein) At1g49490 (a kind gift of Pat Bedinger, Colorado State University) (6) was fused to RBP (7), creating a fusion at residue A20 of the secretory sequence to residue K26 of the RBP protein. A GFP coding sequence was then fused to the C-terminal end of secretory sequence-RBP (ssRBP), and the resulting fusion gene (ssRBP-GFP) was placed under control of the CaMV35S constitutive promoter in the psmGFP vector (8). An *Nde*I site was created next to the *Bam*HI site in the psmGFP vector for C-terminal fusions of proteins to GFP. An internal *Nde*I site (5’*CATATG3’*) in the GFP gene was removed by silent mutagenesis (5’*CACATG3’*) using the QuikChange site directed mutagenesis kit (Stratagene) according to the manufacturer’s instructions, and sequence verified (Macrogen USA, Rockville, MD). The Pex secretory sequence was fused to RBP using overlapping extension PCR (4). The PCR primers introduced a *Bam*HI site at the 5'-end of ssRBP and added a linker encoding 2x Gly-Gly-Ser (GGS) and an *Nde*I site at the 3’-end. The ssRBP fusion was then cloned into the *Bam*HI and *Nde*I sites of our modified psmGFP vector, resulting in pssRBP-smGFP. The Pex secretory sequence was fused to the TNT receptor using the same procedures and the same linker described above for the ssRBP fusion. The ssTNT.R3 gene was then transferred from our modified psmGFP vector using *Bam*HI and *Sac*I to the plant transformation vector pCB302-3 (9), which carries a gene for Basta selection.

***Histidine Kinase Targeting to the Plasma Membrane.*** The signal peptide (amino acid residues M1 to K24) from *Arabidopsis* *FLS2* gene (At5g46330) (10) was fused to the start codon of Trg-PhoR using overlapping extension PCR. A *Bam*HI site added to the 5’-end and *Sgf*I site to the 3’-end. The promoter for the Nopaline Synthase (PNOS) gene was PCR-amplified from pCB302-3, and cloned into pBlueScript SK (Stratagene, La Jolla, CA), resulting in pBS-PNOS. The terminator for Nopaline Synthase (TNOS) was fused to a transcription block (TB) (11) and cloned into pBS-PNOS to make a pBS-PNOS-TNOS-TB cloning vector, with a 5’ *Bam*HI site and a 3’ *Sgf*I site between the promoter and terminator. Fls-Trg-PhoR was ligated into pBS-PNOS-TNOS-TB using *Bam*HI and *Sgf*I. PNOS-Fls-Trg-PhoR-TNOS-TB was PCR-amplified using primers containing *Hind*III sites, digested, and ligated into the *Hind*III site of pCB302-3, which provides Basta resistance (9).

***Assembly of Gene Circuits for a Synthetic Signal Transduction System and Detector Plants.*** For the synthetic signaling system, Fls was added to the optimal Trg-PhoR fusion (DHP8, defined in bacterial testing), cloned into the pBS-PNOS-TNOS-TB vector, and sub-cloned into pCB302-3. The complete synthetic signaling circuit was obtained by co-transforming the above-described plasmid containing ssTNT.R3 and Fls-Trg-PhoR with a previously described plasmid containing the PlantPho system (12), which encodes the signal transmission/transcriptional activator PhoB-VP64 protein expressed under control of the FMV promoter, and the signal receptive PlantPho promoter driving expression of the β-glucuronidase GUS reporter gene (PlantPho::GUS-TNOS). Transgenic plants containing both T-DNAs were obtained by selection with kanamycin and Basta. To assemble the detector gene circuit for the TNT ligand, FMV::PhoB-VP64-TNOS (12) was PCR amplified, ends blunted, and ligated into a *Pme*I site in the pCB302-3 vector containing ssTNT.R3 and PNOS::Fls-Trg-PhoR-TNOS-TB, resulting in the sensing gene circuit. The de-greening readout was incorporated by replacing the 10xN1P promoter driving *POR* diRNA, *Chlorophyllase*, and *RCCR* from de-greening gene circuit #1 (13) with the PlantPho promoter (12), generating the signal-sensitive readout gene circuit. The sensing (kanamycin resistance) and readout (Basta resistance) gene circuits were co-transformed into Arabidopsis and tobacco plants, and transgenic plants containing both T-DNAs were obtained by selection with kanamycin and Basta. All synthetic genes used to make transgenic plants were verified by DNA sequencing (Macrogen USA).

***Transient Assays.*** Sub-cellular localization of the ssRBP-GFP fusion was determined by transient assays using particle bombardment into onion cells. The pssRBP-smGFP vector was bombarded into onion epidermal cells (14) placed on MS agar plates using a PDS-100/He device (BioRad, Hercules, CA), pressure of 1100 psi, and were observed and digitally photographed under a Nikon Diaphot fluorescence microscope after 30-36 hours.

***Testing Plants for Ligand-dependent Induction of β-glucuronidase with Quantitative Fluorometric Assays.*** To quantitatively test the signal transduction gene circuits we used a fluorometric assay and screened fourteen-day-old transgenic *Arabidopsis* plants for TNT-dependent induction of GUS activity. Plants containing the computer re-designed receptor (ssTNT.R3) signal through chimeric transmembrane proteins that consist of the bacterial Trg chemotactic domain fused to a bacterial HK adapted for plants (Fls-Trg-PhoR), and response regulator PhoB-VP64, which activates the synthetic PlantPho promoter (PlantPho::GUS). Whole plants or leaves were incubated for 14-16 hours in water (*control*), or water with TNT at concentrations indicated. Total proteins were extracted by grinding plant tissue in GUS extraction buffer, and fluorometric measurements of GUS activity were performed on a DynaQuant 200 fluorometer (Hoefer Inc., Holliston, MA), as previously described (15). 4-methylumbelliferone (4-MU) was used as a standard. GUS activity was normalized to the total protein content of samples and expressed as nmoles 4-MU mg^-1^ protein h^-1^. Total protein content of samples was measured using the Bradford reagent.

***Screening of Primary Transgenic Plants (T_0_ Leaf Assay) for Ligand-dependent Induction of the De-greening Gene Circuit.***

***Assay.*** We screened T_0_ (primary) transgenic *Arabidopsis* plants for a de-greening response by incubating developmentally equivalent leaves (first and second leaves) from fourteen-day-old plants in water or water plus 100 pM 2,4,6-trinitrotoluene (TNT) (ChemService, West Chester, PA) in 24-well plates. Primary transgenic (T_0_) tobacco plants were screened when plants had 4-6 leaves visible and started to form visible root growth. Tween 20 (Sigma-Aldrich, St. Louis, MO) (0.005%) was added as a wetting agent to the TNT solution prior to leaf incubation. Vacuum was briefly applied and released five times to ensure that the ligand contacted the leaves, and plates were placed on a light rack under the standard growth conditions described above.

***Visible and Fluorometric Responses.*** We documented the ligand-dependent response of plants visually with digital photographs. In addition, because induction of the de-greening gene circuit causes chlorophyll degradation in plastid photosystems (13), we quantified changes in *F_v_/F_m_*, the maximum efficiency of photosystem II. *F_v_/F_m_* changes were calculated by measuring chlorophyll fluorescence with the use of a Fluorcam (Photon Systems Instruments, Brno, Czech Republic) at the indicated time points. Specifically, *F_v_/F_m_* values were calculated using the Quenching Analysis protocol provided with the manufacturer's software. The calculated *F_v_/F_m_* data are presented as graphs, showing average *F_v_/F_m_* values of entire plants, and spatially, where individual pixels of the image display the intensity of *F_v_/F_m_* values, according to the color table shown in each figure, using the manufacturer's software.

***Ligand Detection by Plants.*** To test ligand detection beneath the surface, 21-30 day old transgenic tobacco plants were grown under the conditions described above, then transferred to MS medium supplemented with 50 mg/mL kanamycin, 5 mg/mL Basta, and either 100 nM TNT, 1 μM TNT, 100 nM 2,6-DNT, or 100 nM 2,4-DNT. To ensure that the inducer was only available to the roots, plants were placed in holes made in small Petri dishes resting on the agar, preventing leaves from contacting the medium. These plants were then placed back under growing conditions described above for the duration of the experiment. Leaves from plants that responded to the treatment were excised from the plants, frozen in liquid N_2_, and stored at -80^o^C until RNA extraction. Responsive plants were transferred to soil, allowed to set seed, and analyzed for segregation of the T-DNAs encoding the sensing and de-greening gene circuits.

To test the ability of plants to detect ligands in the air, 21-30 day old transgenic tobacco plants (and wild-type SR1 controls) grown on MS agar plates supplemented with kanamycin and Basta were individually transferred to soil and placed into a sealed plastic bag for 48 hours under the growing conditions described above. Bags were opened and the plants were allowed to acclimate under a Hortilux Super High Pressure Sodium light (Eye Lighting Intl., Mentor, OH) at 115-135 µE.m^-2^.s^-1^, 28^o^C, for 24 hours. Plants were then removed from the bags, grown for additional 48 hours, and placed inside a sealed 170 L glass aquarium in the presence or absence of TNT-NESTT (Non-Explosive Stimulant for Testing and Training, (Van Aken Intl., Rancho Cucamonga, CA). Conditions in the glass aquarium were as follows: 25^o^C, 42% humidity, 135 µE.m^-2^.s^-1^, at the start of the experiment, and 27^o^C, 91.2% humidity, 135 µE.m^-2^.s^-1^, after 48 hours.

***Real-time Quantitative RT-PCR.*** Total RNA was extracted from plants assayed in root sensing or vapor induction experiments using the RNeasy Plant Mini kit (Qiagen), which included a DNAse treatment step to eliminate genomic DNA contamination. Fifty nanograms of total RNA were used in quantitative RT-PCR experiments using the Verso SYBR Green 1-step QRT-PCR kit (Thermo Fisher Scientific, Waltham, MA) in a LightCycler 480 instrument (Roche Applied Science, Indianapolis, IN). Gene-specific primers were designed using PrimerQuest (http://www.idtdna.com/Scitools/Applications/Primerquest) and synthesized by Integrated DNA Technologies (IDT, Coralville, Iowa). Changes in mRNA levels were calculated using the manufacturer’s Relative Quantification software.

***Nuclear Translocation.*** Sub-cellular localization of PhoB-VP64-GFP fusion protein (and the mutant PhoB^D53A^-VP64-GFP) was visualized in roots from six-day-old plants before and after treatment with 10 μM TNT for a minimum of 2 hours, using either a Carl Zeiss LSM 510 META confocal microscope with 20X lens (Plan-Neofluar 0.3NA) in multi-track mode and in-line switching with a scan speed of 5, or an inverted Nikon Diaphot epifluorescence microscope equipped with GFP (excitation 480/20, emission. 510/20) and DAPI (excitation 350/50, emission 460/50) filters. Stacks of 0.5 – 2 µm were taken with Carl Zeiss LSM 510 META using manufacturer-defined filter sets for DAPI and GFP. Nuclei were visualized after staining tissues with 1 ng/µL DAPI (Sigma-Aldrich).

***Statistical Analysis.*** All statistical analyses were performed using JMP software, v. 6.0.3 (SAS Institute, Cary, NC). A *t*-test was used to analyze changes in GUS activity resulting from ligand induction in plants containing control constructs for the Fls-Trg-PhoR system (Fig. 2A). A Paired *t*-test was used for ligand dependent induction of Arabidopsis primary transformants (T_0_) containing the sensing circuit and GUS readout (Fig. 2A). Linear regression of homozygous transgenic plants (containing the sensing and GUS reporter gene circuits) was carried out (Fig. 2B), the dependent variable being the log (measured GUS activity (nmoles 4-MU.mg^-1^ protein.h^-1^)), and the independent variable being log (TNT concentrations + 1) treated as a fixed effect. We used a log-log transformation and found the assumptions of normally distributed residuals were met and added one to the TNT concentrations to account for zero values. A Chi-square (χ^2^) test was conducted to determine the number of T-DNA inserts present in tobacco TNT detector lines (Table S2). All the assumptions of parametric statistics were tested and met.

**References**

1. Clough SJ, Bent AF (1998) Floral dip: a simplified method for Agrobacterium-mediated transformation of Arabidopsis thaliana. *Plant J* 16(6):735-743.

2. Dandekar AM, Fisk HJ (2005) Plant transformation: Agrobacterium-mediated gene transfer. *Methods Mol Biol* 286:35-46.

3. Murashige T, Skoog F (1962) A revised medium for rapid growth and bioassays with tobacco tissue cultures. *Physiologia Plantarum* 15:473-497.

4. Horton RM, Hunt HD, Ho SN, Pullen JK, Pease LR (1989) Engineering hybrid genes without the use of restriction enzymes: gene splicing by overlap extension. *Gene* 77(1):61-68.

5. Crameri A, Whitehorn EA, Tate E, Stemmer WPC (1996) Improved green fluorescent protein by molecular evolution using DNA shuffling. *Nature Biotechnology* 14(3):315-319.

6. Baumberger N*, et al.* (2003) Whole-genome comparison of leucine-rich repeat extensins in Arabidopsis and rice. A conserved family of cell wall proteins form a vegetative and a reproductive clade. *Plant Physiol* 131(3):1313-1326.

7. Binnie RA, Zhang H, Mowbray S, Hermodson MA (1992) Functional mapping of the surface of *Escherichia coli* ribose-binding protein: mutations that affect chemotaxis and transport. *Protein Sci* 1(12):1642-1651.

8. Davis SJ, Vierstra RD (1998) Soluble, highly fluorescent variants of green fluorescent protein (GFP) for use in higher plants. *Plant Mol Biol* 36(4):521-528.

9. Xiang C, Han P, Lutziger I, Wang K, Oliver DJ (1999) A mini binary vector series for plant transformation. *Plant Mol Biol* 40(4):711-717.

10. Gomez-Gomez L, Boller T (2000) FLS2: an LRR receptor-like kinase involved in the perception of the bacterial elicitor flagellin in Arabidopsis. *Mol Cell* 5(6):1003-1011.

11. Padidam M, Cao Y (2001) Elimination of transcriptional interference between tandem genes in plant cells. *Biotechniques* 31(2):328-324.

12. Antunes MS*, et al.* (2009) Engineering Key Components in a Synthetic Eukaryotic Signal Transduction Pathway. *Molecular Systems Biology* 5:270.

13. Antunes MS*, et al.* (2006) A synthetic de-greening gene circuit provides a reporting system that is remotely detectable and has a re-set capacity. *Plant Biotechnology Journal* 4:605-622.

14. Kikkert JR, Vidal JR, Reisch BI (2005) Stable transformation of plant cells by particle bombardment/biolistics. *Methods Mol Biol* 286:61-78.

15. Gallagher SR (1992) in *GUS protocols: using the GUS gene as a reporter of gene expression*, ed Gallagher SR (Academic Press, San Diego), pp 47-59
